# Exploring coping strategies of emergency medical technicians against COVID-19 stress: a qualitative study

**DOI:** 10.3389/fpsyt.2023.1212769

**Published:** 2023-08-02

**Authors:** Mohammad Parvaresh-Masoud, Asma Abdollahyar, Yasamin Molavi-Taleghani, Sahar Salahi, Hojjat Farahmandnia

**Affiliations:** ^1^Department of Emergency Medicine, Paramedical Faculty, Qom University of Medical Sciences, Qom, Iran; ^2^Department of Nursing, Zarand Branch, Islamic Azad University, Zarand, Iran; ^3^Health Management and Economics Research Center, Department of Health Services Management, School of Management and Medical Information Sciences, Isfahan University of Medical Sciences, Isfahan, Iran; ^4^Department of Nursing, Yasuj Branch, Yasuj Islamic Azad University, Yasuj, Iran; ^5^Health in Disasters and Emergencies Research Center, Institute for Futures Studies in Health, Kerman University of Medical Sciences, Kerman, Iran

**Keywords:** coping strategies, emergency medical technicians, COVID-19, stress, qualitative study

## Abstract

**Introduction:**

The COVID-19 pandemic has been shown to cause enormous psychological burden among health care workers, including first responders. However, the psychological well-being of first responders, essential in the fight against COVID-19 pandemic, has often been ignored. This qualitative study aimed to explore the coping strategies used by EMTs to manage stress during the pandemic.

**Methods:**

The research was designed as a qualitative study, utilizing a thematic analysis framework. Semi-structured interviews were conducted with 15 EMTs from a single geographic area between September 2022 and December 2022. The data were analyzed using Braun and Clarke analytic techniques, and this study used Lincoln and Guba’s to assess the reliability of the data.

**Results:**

Four main coping strategies emerged from the data: seeking social support, practicing self-care, utilizing coping mechanisms, and finding meaning and purpose in work. EMTs sought support from both professional and personal sources, engaged in physical and emotional self-care, used humor and distraction as coping mechanisms, and found meaning and purpose in helping others and growing as professionals.

**Conclusion:**

Based on the extracted results from the study on EMTs in Iran, it can be concluded that the importance of social support cannot be overstated, as it serves as a crucial buffer against the negative effects of stress and trauma. The findings suggest that addressing the social and emotional needs of EMTs is important for promoting their mental health and well-being during the pandemic.

## Introduction

The COVID-19 pandemic has brought unprecedented challenges to the healthcare industry worldwide, and emergency medical technicians (EMTs) are among the frontline workers who are at the forefront of responding to the crisis (1). The mental health of healthcare workers has been a growing concern during the COVID-19 pandemic. Studies have reported high levels of stress, anxiety, and depression among healthcare workers, including EMTs. Factors contributing to the high levels of stress include the fear of contracting the virus, concerns for family and friends, a shortage of personal protective equipment (PPE), and an increased workload (2, 3). The pandemic has also led to the adoption of new work practices and policies, which have resulted in additional stressors for EMTs (2, 4, 5).

The COVID-19 pandemic has had a significant impact on healthcare workers worldwide, especially those on the frontlines such as emergency medical technicians (EMTs). As the number of COVID-19 cases continues to rise, it is crucial to understand the coping strategies used by EMTs to manage stress and promote their well-being. Previous research has identified social support, self-care, and coping mechanisms as effective strategies for managing stress among EMTs (6–8). However, there is limited research on the coping strategies used by EMTs during the COVID-19 pandemic. Therefore, the aim of this study was to explore the coping strategies used by EMTs to manage stress during the pandemic.

Iran has been one of the countries hit hard by the COVID-19 pandemic, and the healthcare system has faced significant challenges in responding to the crisis (9). The Iranian government has taken measures to address the pandemic, including closing down schools and universities, implementing travel restrictions, and increasing the availability of PPE (10). Despite these efforts, the healthcare system has been under strain, and EMTs have faced significant challenges in managing their work-related stress. In Iran, as in many other countries, EMTs have been working tirelessly to provide emergency care and transport patients during the COVID-19 pandemic.

The purpose of this study is to explore the coping strategies used by EMTs in Iran to manage stress during the COVID-19 pandemic. The study uses a qualitative approach to gather data from EMTs in Iran, which allows for an in-depth exploration of their experiences and coping strategies. By gaining a better understanding of the coping strategies used by EMTs, this study can contribute to the development of tailored interventions and support programs to promote their well-being during this challenging time.

## Methods

### Research design

The research was designed as a qualitative study, utilizing a thematic analysis framework. The aim of the study was to investigate the protective factors and coping mechanisms related to COVID-19 among EMTs in Iran. Thematic analysis is a systematic and interpretive method used to identify and analyze patterns within the data, resulting in a comprehensive understanding of the phenomenon under investigation (11). This approach does not aim to create a theory but rather to identify meaningful themes (12). To ensure the reliability of the findings, both authors independently read the data multiple times to extract and code any themes that were relevant to the research objective.

### Participants

A purposeful sampling method was used to recruit a sample of 15 EMTs who were directly involved in the care of COVID-19 patients between September 2022 and December 2022. The inclusion criteria for the study were EMTs who had direct contact with COVID-19 patients, including those who transported patients from their homes or local hospitals to COVID-19 wards or centers, as well as volunteers who expressed interest in participating. Participants who did not consent to participate or withdrew from the study during or within a week after the interview were excluded. Initially, 20 participants expressed interest in participating, but five of them declined for personal reasons. The final sample consisted of 15 participants, who were assured of confidentiality and anonymity. Prior to the interviews, rapport building sessions were conducted to establish a connection with the participants and explain the purpose of the study.

### Data collection

The data collection method was semi-structured interviews. The interview plan was designed after reviewing past texts, discussing it with qualitative research experts, and conducting some preliminary interviews with a number of emergency medical technicians. Both the researcher and the interview subjects felt at ease discussing their thoughts because the interviews were done in Persian. The interviews were conducted one-on-one in a welcoming environment where the participants felt at ease. The interviewer first obtained the overall perspective of the emergency medical technicians on COVID-19 in a province of Iran and their understanding of the disease caused by this virus through general questions such as “How do you see COVID-19 in Iran? What is your understanding of this disease caused by the virus?” The interviewer then asked them to explain the challenges of caring for patients with COVID-19. The duration of the interviews ranged from 35 to 75 min, and was different for each participant based on their response time. The interviews continued until data saturation was reached. Data saturation refers to the point where collecting more data would result in repeating previously collected data and not obtaining new information (13). In this study, data saturation was achieved after 12 interviews, and to ensure that no new data was obtained, three additional interviews were conducted.

### Data analysis

In order to analyze the data obtained in the study, Braun and Clarke (14) have suggested several transcription methods that are tailored to different analytical approaches. In this particular study, an orthographic transcription was employed, which involved creating a verbatim record of all verbal and nonverbal expressions that were observed. Both authors carried out independent analyses of the data. They thoroughly read through the transcripts several times, condensed the material, and derived meaningful statements, themes, and sub-themes. Any disagreements or discrepancies between the two authors were resolved through mutual discussion, and a consensus was reached. To maintain the anonymity of the participants, each individual was assigned a numerical code during the analysis process, which allowed for the data to be analyzed without revealing their identities. It should be noted that this analysis was conducted without reference to any previous studies on the topic, in order to ensure a completely independent and unbiased analysis of the data.

### Trustworthiness

For reliability and validity tests, this study used Lincoln and Guba’s recommendations (15). The validity, verifiability, reproducibility, and provability axes serve as the foundation for the Lincoln and Guba evaluation technique for qualitative research validation. These standards are frequently applied for validating qualitative research. This is why these two academics are more well-known within studies for the topic of validity and reliability in qualitative research. Four requirements creditability, dependency, conformability, and transferability—are allegedly necessary to provide reliability. To increase the dependability of the data, the researchers spent 11 months interacting with the data and the surrounding area while continuously making observations and accumulating field notes. The trustworthiness of the data was assessed using peer-check processes. To make sure that the study team had a thorough discussion of the recently obtained data, monthly peer checks were carried out. Data conformability was evaluated using background knowledge, variables such as the researchers’ interests in the pertinent themes, document handling, and others. The context of the interviews, codes, and extracted categories were checked by the study team, additional specialists, and three top experts in qualitative research. By using maximum variation sampling, the researchers were able to collect a wide range of remarks, observations, and interpretations.

## Results

The participants were all male, and their ages ranged from 28 to 38 years old, with a mean age of 31.87 ± 2.82. Their working experience ranged from 2 to 12 years, with a mean of 6.53 ± 2.44. All participants had at least an associate’s degree. Table 1 presents the participants’ characteristics in more detail. The findings revealed several themes related to the psychological impact of COVID-19 on EMTs, their coping strategies, and their challenges. The thematic analysis of the data revealed four main coping strategies used by EMTs to manage stress during the COVID-19 pandemic: (1) seeking social support, (2) practicing self-care, (3) utilizing coping mechanisms, and (4) finding meaning and purpose in their work. Each of these main themes included several sub-themes that provided more detail on the coping strategies used by EMTs, shows in Table 2.

**Table 1:** Characteristics of the study participants (*n* = 15).

Participant	Age (years)	Working experience (years)	Education level
P1	32	7	Bachelor’s degree
P2	29	3	Associate’s degree
P3	30	5	Bachelor’s degree
P4	31	4	Bachelor’s degree
P5	33	8	Bachelor’s degree
P6	35	10	Bachelor’s degree
P7	34	9	Bachelor’s degree
P8	28	2	Associate’s degree
P9	36	11	Bachelor’s degree
P10	31	4	Bachelor’s degree
P11	32	7	Bachelor’s degree
P12	30	5	Bachelor’s degree
P13	33	8	Bachelor’s degree
P14	38	12	Bachelor’s degree
P15	31	4	Bachelor’s degree

**Table 2:** Coping strategies used by EMTs during COVID-19 pandemic.

Main theme	Sub-themes
Seeking social support	Professional support
	Personal support
Practicing self-care	Physical self-care
	Emotional self-care
	Time management
Utilizing coping mechanisms	Humor
	Distraction
Finding meaning and purpose in work	Helping others
	Professional growth

### Seeking social support

Securing social support can be an effective strategy for managing stress during difficult times. By seeking professional support from colleagues and supervisors at work and personal support from family and friends outside of work, individuals can receive the help and assistance they need to manage stress and maintain their mental health.

#### Professional support

Professional support refers to the help, guidance, and advice that individuals receive from trained professionals in their field of work. This can include colleagues, supervisors, mentors, and other professionals who have experience in dealing with similar situations. P6 shared: *“I spoke to my supervisor and colleagues about the stress I was experiencing and they helped me develop coping strategies to manage it.”*

#### Personal support

Personal support refers to the help and assistance that individuals receive from their family and friends outside of work. This can include emotional support, practical support, and social support. P2 shared: *“My family has been a great source of support for me during these difficult times. They listen to me when I need to talk and help me relax when I’m feeling stressed.”*

### Practicing self-care

Practicing self-care has been identified as an effective coping strategy for managing stress among healthcare workers, including EMTs, during the COVID-19 pandemic. By prioritizing physical and emotional self-care and effectively managing their time, EMTs can better manage the stress and demands of their work during this challenging time.

#### Physical self-care

Physical self-care involves engaging in activities that promote physical well-being, such as exercise and healthy eating. One EMT participant in the study described the importance of physical self-care: “I make sure to eat healthy and exercise, even if it’s just for a short time. It helps me feel better and more energized for work” (p. 3). Another participant stated, *“I try to make sure I’m taking care of my physical needs, so I can be better prepared to handle the demands of my job”* (p. 8).

#### Emotional self-care

Emotional self-care involves engaging in activities that promote emotional well-being, such as mindfulness and journaling. One EMT participant in the study described the importance of emotional self-care: “I try to take some time each day to check in with myself and process my emotions. It helps me stay grounded and focused on my work” (p. 9). Another participant stated, *“Journaling has been really helpful for me. It allows me to process my thoughts and feelings in a safe space”* (p. 7).

#### Time management

Time management involves prioritizing one’s time and setting boundaries to manage their workload. One EMT participant in the study described the importance of time management: “I try to prioritize my tasks and set realistic goals for myself. It helps me feel more in control and reduces my stress levels” (p. 11). Another participant stated, *“Setting boundaries with work is really important. I try to make sure I’m taking breaks and not overworking myself”* (p. 10).

### Utilizing coping mechanisms

Coping mechanisms are one of the most commonly used strategies to manage stress among EMTs. The data from this study revealed two sub-themes under the main theme of utilizing coping mechanisms, including humor and distraction.

#### Humor

EMTs used humor as a coping mechanism to deal with the stress of their job. P2 stated, *“We have to keep things light sometimes, because if we do not, it’s just too much to handle.”*

#### Distraction

EMTs also utilized distraction as a coping mechanism to manage stress. Distraction refers to engaging in activities that take one’s mind off of the stressor or situation causing the stress. Participants reported engaging in activities such as reading, watching TV, and playing video games as a way to distract themselves from the stress of their work.

One participant described the use of distraction as follows: *“When I’m off the clock, I try to do things that take my mind off of work. I’ll watch TV or play video games, just to give myself a break from everything”* (p. 5).

### Finding meaning and purpose in work

One of the coping strategies identified by EMTs in managing stress during the COVID-19 pandemic was finding meaning and purpose in their work. This involved seeing their work as a way to contribute to society and help those in need. Two sub-themes emerged under this main theme: helping others and professional growth.

#### Helping others

EMTs found meaning and purpose in their work by helping others during the pandemic. One participant stated, *“It feels good to know that I am helping people who really need it. That’s what keeps me going”* (p. 4). This sub-theme highlights the importance of altruism and the intrinsic motivation that comes from helping others.

#### Professional growth

EMTs also found meaning and purpose in their work by learning and growing as professionals. This involved taking on new challenges and developing new skills. As one participant stated, *“I feel like I am constantly learning and growing in this job, and that makes me feel good about what I am doing”* (p. 9). This sub-theme highlights the importance of professional development and the sense of accomplishment that comes from developing one’s skills and knowledge.

## Discussion

This study aimed to explore the coping strategies used by EMTs in Iran to manage stress during the COVID-19 pandemic. The results of the thematic analysis showed that participants used and suggested a variety of coping mechanisms to manage the stress and anxiety brought on by the COVID-19 epidemic. The findings suggest that seeking social support, practicing self-care, utilizing coping mechanisms, and finding meaning and purpose in work are effective strategies for managing stress among EMTs.

The first theme identified in this study was seeking social support, which included both professional and personal support. This finding is consistent with previous research on coping strategies used by healthcare workers during pandemics. For example, a study conducted by Beck and Daniels found that seeking social support was an effective coping strategy for healthcare workers during the COVID-19 pandemic (16). Similarly, a study by Zhang et al. found that social support was a critical factor in reducing psychological distress among healthcare workers during the COVID-19 pandemic (17). These findings suggest that seeking social support is a universal coping strategy that is effective in managing stress among healthcare workers during pandemics. The study also found that professional support, such as support from colleagues and supervisors in the workplace, was an important source of support for healthcare workers. This finding is consistent with previous research that highlights the importance of supportive work environments in promoting the well-being of healthcare workers. For example, a study by Martin et al. found that supportive work environments were associated with lower levels of burnout and higher levels of job satisfaction among physicians (18). Similarly, a study by Tomlin et al. found that supportive work environments were associated with lower levels of burnout and higher levels of engagement among health care providers (19). These findings suggest that creating a supportive work environment that prioritizes the well-being of healthcare workers is an important strategy for promoting the mental health and well-being of healthcare workers during pandemics and other emergency situations.

The second theme identified in this study was practicing self-care, which included physical self-care, emotional self-care, and time management. These findings are consistent with previous research that highlights the importance of self-care strategies in managing stress and promoting well-being among healthcare workers. For example, a study by Ross et al. found that self-care behaviors, such as exercise and healthy eating, were associated with lower levels of burnout among nurses (20). Similarly, a study showed that self-care activities, such as mindfulness and relaxation techniques, were effective in reducing stress and improving well-being among pediatric critical care nurses and physicians (21). The study found that physical self-care, such as exercise and healthy eating, was an effective strategy for managing stress among healthcare workers. This finding is consistent with previous research that suggests that physical self-care is an important component of overall well-being. For example, a study showed that regular exercise was associated with lower levels of depression and anxiety among healthcare workers (22). Additionally, the study found that emotional self-care, such as mindfulness and journaling, was an effective strategy for managing stress. This finding is consistent with previous research that suggests that emotional self-care can help healthcare workers manage the emotional demands of their work and reduce stress (23). The study also identified time management as an important self-care strategy for healthcare workers. This finding is consistent with previous research that suggests that time management skills can help older adult caregivers manage their workload and reduce stress (24). Overall, these findings suggest that practicing self-care strategies, including physical and emotional self-care and time management, can be effective in managing stress and promoting well-being among healthcare workers during pandemics and other emergency situations.

The third theme identified in this study was utilizing coping mechanisms, which included humor and distraction. The study found that utilizing these coping mechanisms was an effective strategy for managing stress among healthcare workers during the COVID-19 pandemic. These findings are consistent with previous research that suggests that utilizing coping mechanisms can be an effective strategy for managing stress and promoting well-being among healthcare workers (25, 26). The study found that humor was an effective coping mechanism for healthcare workers during the COVID-19 pandemic. This finding is consistent with previous research that suggests that humor can help healthcare workers manage the emotional demands of their work and reduce stress (27). Additionally, the study found that distraction was an effective coping mechanism for managing stress among healthcare workers. This finding is consistent with previous research that suggests that distraction can help healthcare workers manage stress and reduce burnout (28). Overall, these findings suggest that utilizing coping mechanisms, such as humor and distraction, can be effective in managing stress and promoting well-being among healthcare workers during pandemics and other emergency situations. However, it is important to note that not all coping mechanisms may be equally effective for all individuals. Healthcare workers may need to experiment with different coping mechanisms to find the strategies that work best for them. Additionally, healthcare organizations and managers may need to provide resources and support to help healthcare workers develop and utilize effective coping mechanisms during times of crisis.

The fourth theme identified in this study was finding meaning and purpose in work, which included helping others and professional growth. The study found that finding meaning and purpose in work was an effective strategy for managing stress and promoting well-being among healthcare workers during the COVID-19 pandemic. These findings are consistent with previous research that suggests that finding meaning and purpose in work can help healthcare workers manage stress and reduce burnout (29). The study found that helping others was an important source of meaning and purpose for healthcare workers. This finding is consistent with previous research that suggests that helping others is a key motivator for healthcare workers and can contribute to overall job satisfaction (30). Additionally, the study found that professional growth, such as continuing education and skill development, was an important source of meaning and purpose for healthcare workers. This finding is consistent with previous research that suggests that professional growth is an important component of job satisfaction and can contribute to overall well-being (31). Overall, these findings suggest that finding meaning and purpose in work, through helping others and professional growth, can be an effective strategy for managing stress and promoting well-being among healthcare workers during pandemics and other emergency situations. Healthcare organizations and managers may need to provide resources and support to help healthcare workers find meaning and purpose in their work and develop opportunities for professional growth, particularly during times of crisis.

## Conclusion

In conclusion, this study identified several coping strategies that were effective in managing stress and promoting well-being among healthcare workers during the COVID-19 pandemic. Seeking social support, practicing self-care, utilizing coping mechanisms, and finding meaning and purpose in work were identified as important strategies for managing stress and promoting well-being. These findings are consistent with previous research on coping strategies used by healthcare workers during pandemics. The study also highlights the importance of creating a supportive work environment that prioritizes the well-being of healthcare workers and provides resources and support to help them develop and utilize effective coping strategies. Overall, these findings have important implications for healthcare organizations and managers in promoting the mental health and well-being of healthcare workers during pandemics and other emergency situations.

## Data availability statement

The original contributions presented in the study are included in the article/supplementary material, further inquiries can be directed to the corresponding authors.

## Ethics statement

A qualitative study with Reg. No. 402000150 was approved by ethical committee of Kerman University of Medical Sciences. All methods were performed in accordance with the relevant guidelines and regulations; this article does not contain any studies with animals performed by any of the authors. Informed consent was obtained from all individual participants included in the study written informed consent was obtained from individual participants. Confidentiality and anonymity of the participants were ensured by coding of the questioners. Study participants were informed clearly about their freedom to opt out of the study at any point of time without justifying for doing so.

## Author contributions

HF and AA conceived the concept and design of the study. MP-M and YM-T conducted the survey. HF was involved in data analysis and manuscript writing. HF and SS supervised the study and critically reviewed the manuscript. All the authors read reviewed the final manuscript.

## Conflict of interest

The authors declare that the research was conducted in the absence of any commercial or financial relationships that could be construed as a potential conflict of interest.

## Publisher’s note

All claims expressed in this article are solely those of the authors and do not necessarily represent those of their affiliated organizations, or those of the publisher, the editors and the reviewers. Any product that may be evaluated in this article, or claim that may be made by its manufacturer, is not guaranteed or endorsed by the publisher.
